# Supplementary material for: Changing the incentive structure of social media platforms to halt the spread of misinformation
Source: eLife. 2023 Jun 6;12:e85767. doi: 10.7554/eLife.85767 (PMC10259455; doi:10.7554/eLife.85767)
Supplement: Supplementary file 1. [file elife-85767-supp1.docx]

**Supplementary file 1. Discernment of reactions (Experiment 1)**.

| **Discernment** | **df** | **F-value** | **p-value** |
| --- | --- | --- | --- |
| including demographics |  |  |  |
| **Type of Reaction** | (1,104) | 95.832 | <0.001 |
| **Valence of Reaction** | (1,105) | 17.33 | <0.001 |
| **Gender** | (1,101) | 8.698 | 0.004 |
| **Political Orientation** | (1,101) | 26.928 | <0.001 |
| **Ethnicity** | (1,101) | 0.276 | 0.601 |
| **Age** | (1,101) | 0.884 | 0.349 |
| **Type of Reaction x Political Orientation** | (1,104) | 24.084 | <0.001 |
| including valence x reaction |  |  |  |
| **Type of Reaction** | (1,106) | 80.936 | <0.001 |
| **Valence of Reaction** | (1,106) | 18.26 | <0.001 |
| **Type of Reaction x Valence of Reaction** | (1,106) | 51.489 | <0.001 |

The interaction of type and valence of reaction is characterized by participants using the ‘distrust’ reaction button (M=0.157, SE=0.008) in a more discerning manner than the ‘trust’ reaction button (M=0.099, SE=0.008; t(106)=9.338, p<0.001, Cohen’s d=0.903), but the ‘like’ reaction button (M=0.06, SE=0.008) in a more discerning manner than the ‘dislike’ button (M=0.034, SE=0.008; t(106)=3.474, p<0.001, Cohen’s d=0.336).
