# Supplementary material for: Changing the incentive structure of social media platforms to halt the spread of misinformation
Source: eLife. 2023 Jun 6;12:e85767. doi: 10.7554/eLife.85767 (PMC10259455; doi:10.7554/eLife.85767)
Supplement: Supplementary file 2. [file elife-85767-supp2.docx]

**Supplementary file 2. % Reactions out of all posts. (Experiment 1).**

| **% Reactions** | **df** | **F-value** | **p-value** |
| --- | --- | --- | --- |
| including demographics |  |  |  |
| **Type of Reaction** | (1,104) | 36.672 | <0.001 |
| **Valence of Reaction** | (1,105) | 13.964 | <0.001 |
| **Gender** | (1,101) | 0.891 | 0.347 |
| **Political Orientation** | (1,101) | 0.939 | 0.335 |
| **Ethnicity** | (1,101) | 0.139 | 0.71 |
| **Age** | (1,101) | 9.519 | 0.003 |
| **Type of Reaction x Political Orientation** | (1,104) | 0.062 | 0.803 |
| including valence x reaction |  |  |  |
| **Type of Reaction** | (1,106) | 37.785 | <0.001 |
| **Valence of Reaction** | (1,106) | 14.891 | <0.001 |
| **Type of Reaction x Valence of Reaction** | (1,106) | 0.19 | 0.664 |
