# Supplementary material for: Changing the incentive structure of social media platforms to halt the spread of misinformation
Source: eLife. 2023 Jun 6;12:e85767. doi: 10.7554/eLife.85767 (PMC10259455; doi:10.7554/eLife.85767)
Supplement: Supplementary file 3. [file elife-85767-supp3.docx]

**Supplementary file 3. Discernment of reactions (Experiment 4**, including type x valence of reaction interaction**)**.

| **Discernment** | **df** | **F-value** | **p-value** |
| --- | --- | --- | --- |
| **Type of Reaction** | (1,49) | 51.996 | <0.001 |
| **Valence of Reaction** | (1,49) | 7.147 | 0.01 |
| **Type of Reaction * Valence of Reaction** | (1,49) | 71.625 | <0.001 |

The interaction is characterized by participants using the ‘distrust’ reaction button (M=0.157, SE=0.014) in a more discerning manner than the ‘trust’ reaction button (M=0.066, SE=0.01; t(49)=7.192, p<0.001, Cohen’s d=1.017), but the ‘like’ reaction button (M=0.053, SE=0.008) in a more discerning manner than the ‘dislike’ button (M=0.007, SE=0.008; t(49)=4.407, p<0.001, Cohen’s d=0.623).
