# Supplementary material for: Changing the incentive structure of social media platforms to halt the spread of misinformation
Source: eLife. 2023 Jun 6;12:e85767. doi: 10.7554/eLife.85767 (PMC10259455; doi:10.7554/eLife.85767)
Supplement: Supplementary file 4. [file elife-85767-supp4.docx]

**Supplementary file 4. % true and false posts shared out of all true or false posts in that feedback condition (Experiment 2).**

| **Feedback Condition** | **%True Posts Shared out of all true posts (SE)** | **%False Posts Shared out of all false posts (SE)** | **% of True Posts Shared Minus % False Posts Shared** |
| --- | --- | --- | --- |
| **Trust** | 40 (3.419) | 21 (2.423) | 18 (2.592) |
| **Like** | 37 (2.536) | 29 (2.233) | 8 (2.083) |
| **Distrust** | 41 (3.192) | 24 (2.7) | 18 (2.646) |
| **Dislike** | 40 (3.715) | 31 (3.343) | 9 (3.92) |
| **Baseline** | 32 (3.537) | 23 (2.817) | 8 (2.498) |
