# Supplementary material for: Changing the incentive structure of social media platforms to halt the spread of misinformation
Source: eLife. 2023 Jun 6;12:e85767. doi: 10.7554/eLife.85767 (PMC10259455; doi:10.7554/eLife.85767)
Supplement: Supplementary file 5. [file elife-85767-supp5.docx]

**Supplementary file 5. Discernment of sharing behavior (Experiment 2).**

| **Discernment** | **df** | **F-value** | **p-value** |
| --- | --- | --- | --- |
| including demographics |  |  |  |
| **Intercept** | (1,278) | 15.286 | <0.001 |
| **Type of Feedback** | (1,278) | 14.908 | <0.001 |
| **Valence of Feedback** | (1,278) | 0.105 | 0.746 |
| **Gender** | (1,278) | 2.977 | 0.086 |
| **Political Orientation** | (1,278) | 66.606 | <0.001 |
| **Ethnicity** | (1,278) | 0.688 | 0.408 |
| **Age** | (1,278) | 0.071 | 0.791 |
| **Type of Feedback x Political Orientation** | (1,278) | 3.012 | 0.051 |
| including valence x reaction |  |  |  |
| **Intercept** | (1,311) | 105.905 | 0.001 |
| **Type of Feedback** | (1, 311) | 12.238 | <0.001 |
| **Valence of Feedback** | (1, 311) | 0.012 | 0.913 |
| **Type of Feedback x Valence of Feedback** | (1,311) | 0.199 | 0.656 |
