# Supplementary material for: Changing the incentive structure of social media platforms to halt the spread of misinformation
Source: eLife. 2023 Jun 6;12:e85767. doi: 10.7554/eLife.85767 (PMC10259455; doi:10.7554/eLife.85767)
Supplement: Supplementary file 6. [file elife-85767-supp6.docx]

**Supplementary file 6. % posts shared out of all posts (Experiment 2).**

| **% posts shared** | **df** | **F-value** | **p-value** |
| --- | --- | --- | --- |
| including demographics |  |  |  |
| **Intercept** | (1,278) | 78.161 | <0.001 |
| **Type of Feedback** | (1,278) | 1.031 | 0.311 |
| **Valence of Feedback** | (1,278) | 1.219 | 0.270 |
| **Gender** | (1,278) | 4.479 | 0.035 |
| **Political Orientation** | (1,278) | 1.518 | 0.219 |
| **Ethnicity** | (1,278) | 2.341 | 0.127 |
| **Age** | (1,278) | 0.032 | 0.858 |
| **Type of Feedback x Political Orientation** | (1,278) | 0.117 | 0.890 |
| including valence x reaction |  |  |  |
| **Intercept** | (1,311) | 701.419 | <0.001 |
| **Type of Feedback** | (1, 311) | 1.533 | 0.217 |
| **Valence of Feedback** | (1, 311) | 0.741 | 0.39 |
| **Type of Feedback x Valence of Feedback** | (1, 311) | 0 | 0.986 |
