# Supplementary material for: Changing the incentive structure of social media platforms to halt the spread of misinformation
Source: eLife. 2023 Jun 6;12:e85767. doi: 10.7554/eLife.85767 (PMC10259455; doi:10.7554/eLife.85767)
Supplement: Supplementary file 7. [file elife-85767-supp7.docx]

**Supplementary file 7. Discernment of sharing behavior (Experiment 5).**

| **Discernment** | **df** | **F-value** | **p-value** |
| --- | --- | --- | --- |
| **Intercept** | (1,256) | 157.841 | <0.001 |
| **Type of Feedback** | (1,256) | 8.08 | 0.005 |
| **Valence of Feedback** | (1,256) | 0.009 | 0.927 |
| **Type of Feedback * Valence of Feedback** | (1,256) | 0.001 | 0.982 |
