# Supplementary material for: Changing the incentive structure of social media platforms to halt the spread of misinformation
Source: eLife. 2023 Jun 6;12:e85767. doi: 10.7554/eLife.85767 (PMC10259455; doi:10.7554/eLife.85767)
Supplement: Supplementary file 8. [file elife-85767-supp8.docx]

**Supplementary file 8. Belief Accuracy (Experiment 2).**

| **Belief Accuracy** | **df** | **F-value** | **p-value** |
| --- | --- | --- | --- |
| including demographics |  |  |  |
| **Intercept** | (1,278) | 78.161 | <0.001 |
| **Type of Feedback** | (1,278) | 7.679 | 0.006 |
| **Valence of Feedback** | (1,278) | 0.386 | 0.535 |
| **Gender** | (1,278) | 2.112 | 0.147 |
| **Political Orientation** | (1,278) | 7.593 | 0.006 |
| **Ethnicity** | (1,278) | 3.984 | 0.047 |
| **Age** | (1,278) | 0.038 | 0.845 |
| **Type of Feedback x Political Orientation** | (1,278) | 0.171 | 0.843 |
| including valence x reaction |  |  |  |
| **Intercept** | (1,311) | 7536.676 | <0.001 |
| **Type of Feedback** | (1,311) | 8.847 | 0.003 |
| **Valence of Feedback** | (1,311) | 0.948 | 0.331 |
| **Type of Feedback x Valence of Feedback** | (1,311) | 0.323 | 0.57 |
