# Supplementary material for: Changing the incentive structure of social media platforms to halt the spread of misinformation
Source: eLife. 2023 Jun 6;12:e85767. doi: 10.7554/eLife.85767 (PMC10259455; doi:10.7554/eLife.85767)
Supplement: Supplementary file 9. [file elife-85767-supp9.docx]

**Supplementary file 9. Belief Accuracy (Experiment 5).**

| **Belief Accuracy** | **df** | **F-value** | **p-value** |
| --- | --- | --- | --- |
| **Intercept** | (1,257) | 6727.546 | <0.001 |
| **Type of Feedback** | (1,257) | 4.151 | 0.043 |
| **Valence of Feedback** | (1,257) | 2.591 | 0.109 |
| **Type of Feedback * Valence of Feedback** | (1,257) | 0.013 | 0.909 |
