# Supplementary material for: Changing the incentive structure of social media platforms to halt the spread of misinformation
Source: eLife. 2023 Jun 6;12:e85767. doi: 10.7554/eLife.85767 (PMC10259455; doi:10.7554/eLife.85767)
Supplement: Supplementary file 10. [file elife-85767-supp10.docx]

**Supplementary file 10. Discernment of sharing behavior (Experiment 3).**

| **Discernment** | **df** | **F-value** | **p-value** |
| --- | --- | --- | --- |
| including demographics |  |  |  |
| **Intercept** | (1,381) | 1.231 | 0.268 |
| **Type of Feedback** | (1,381) | 11.028 | <0.001 |
| **Gender** | (1,381) | 1.357 | 0.259 |
| **Political Orientation** | (1,381) | 6.233 | 0.013 |
| **Ethnicity** | (1,381) | 0.169 | 0.682 |
| **Age** | (1,381) | 0.002 | 0.968 |
| **Type of Feedback x Political Orientation** | (1,381) | 1.524 | 0.219 |
|  |  |  |  |
| **Intercept** | (1,400) | 42.658 | <0.001 |
| **Type of Feedback** | (1, 400) | 11.416 | <0.001 |
