# Supplementary material for: Changing the incentive structure of social media platforms to halt the spread of misinformation
Source: eLife. 2023 Jun 6;12:e85767. doi: 10.7554/eLife.85767 (PMC10259455; doi:10.7554/eLife.85767)
Supplement: Supplementary file 11. [file elife-85767-supp11.docx]

**Supplementary file 11. % posts shared out of all posts (Experiment 3).**

| **% posts shared** | **df** | **F-value** | **p-value** |
| --- | --- | --- | --- |
| including demographics |  |  |  |
| **Intercept** | (1,381) | 41.384 | <0.001 |
| **Type of Feedback** | (1,381) | 8.97 | <0.001 |
| **Gender** | (1,381) | 0.614 | 0.542 |
| **Political Orientation** | (1,381) | 3.98 | 0.047 |
| **Ethnicity** | (1,381) | 4.732 | 0.03 |
| **Age** | (1,381) | 1.146 | 0.285 |
| **Type of Feedback x Political Orientation** | (1,381) | 0.342 | 0.71 |
|  |  |  |  |
| **Intercept** | (1,400) | 932.702 | <0.001 |
| **Type of Feedback** | (1, 400) | 8.897 | <0.001 |
