# Supplementary material for: Changing the incentive structure of social media platforms to halt the spread of misinformation
Source: eLife. 2023 Jun 6;12:e85767. doi: 10.7554/eLife.85767 (PMC10259455; doi:10.7554/eLife.85767)
Supplement: Supplementary file 12. [file elife-85767-supp12.docx]

**Supplementary file 12. Belief Accuracy (Experiment 3).**

| **Belief Accuracy** | **df** | **F-value** | **p-value** |
| --- | --- | --- | --- |
| including demographics |  |  |  |
| **Intercept** | (1,381) | 1123.65 | <0.001 |
| **Type of Feedback** | (1,381) | 3.248 | 0.04 |
| **Gender** | (1,381) | 14.164 | <0.001 |
| **Political Orientation** | (1,381) | 14.749 | <0.001 |
| **Ethnicity** | (1,381) | 3.486 | 0.063 |
| **Age** | (1,381) | 25.786 | <0.001 |
| **Type of Feedback x Political Orientation** | (1,381) | 0.301 | 0.74 |
|  |  |  |  |
| **Intercept** | (1,400) | 18657.083 | <0.001 |
| **Type of Feedback** | (1,400) | 1.043 | 0.353 |
