# Supplementary material for: Changing the incentive structure of social media platforms to halt the spread of misinformation
Source: eLife. 2023 Jun 6;12:e85767. doi: 10.7554/eLife.85767 (PMC10259455; doi:10.7554/eLife.85767)
Supplement: Supplementary file 13. [file elife-85767-supp13.docx]

**Supplementary file 13. Mean difference in posterior distributions and 95% HDI Comparison in Experiment 2.**

| **Estimate** | **‘(Dis)Trust’ minus Baseline** | **‘(Dis)Trust’ minus ‘(Dis)Like’** | **‘(Dis)Like’ minus Baseline** |
| --- | --- | --- | --- |
| **Distance between Decision Thresholds (α)** | 0.25 [0.105; 0.398] | 0.03 [-0.13; 0.188] | 0.22 [0.102; 0.339] |
| **Non-Decision Time (t0)** | -0.34 [-0.644; -0.039] | -0.255 [-0.557; 0.045] | -0.089 [-0.291; 0.107] |
| **Starting Point (z)** | -0.016 [-0.032; 0.001] | -0.011 [-0.025; 0.003] | -0.005 [-0.02; 0.009] |
| **Drift Rate (v)** | 0.118 [0.041; 0.195] | 0.115 [0.048; 0.183] | 0.002[-0.075; 0.08] |
