# Supplementary material for: Changing the incentive structure of social media platforms to halt the spread of misinformation
Source: eLife. 2023 Jun 6;12:e85767. doi: 10.7554/eLife.85767 (PMC10259455; doi:10.7554/eLife.85767)
Supplement: Supplementary file 14. [file elife-85767-supp14.docx]

**Supplementary file 14. Mean difference in posterior distributions and 95% HDI Comparison in Experiment 3.**

| **Estimate** | ‘(Dis)Trust’ minus Baseline | ‘(Dis)Trust’ minus ‘(Dis)Like’ | ‘(Dis)Like’ minus Baseline |
| --- | --- | --- | --- |
| **Distance between Decision Thresholds (α)** | -0.029 [-0.15; 0.091] | 0.002 [-0.114; 0.119] | -0.031 [-0.155; 0.095] |
| **Non-Decision Time (t0)** | 0.176 [-0.03; 0.381] | 0.025 [-0.177; 0.224] | 0.151 [-0. 057; 0.354] |
| **Starting Point (z)** | -0.011 [-0.027; 0.005] | -0.012 [-0.028; 0.003] | 0.001 [-0.015; 0.016] |
| **Drift Rate (v)** | 0.114 [0.061; 0.167] | 0.083 [0.032; 0.135] | 0.031 [-0.0164; 0.079] |
