# Supplementary material for: Changing the incentive structure of social media platforms to halt the spread of misinformation
Source: eLife. 2023 Jun 6;12:e85767. doi: 10.7554/eLife.85767 (PMC10259455; doi:10.7554/eLife.85767)
Supplement: Supplementary file 15. [file elife-85767-supp15.docx]

**Supplementary file 15. Group estimates for DDM in Experiment 5.**

| **Estimate** | **Baseline** | **‘(Dis)Like’** | **‘(Dis)Trust’** |
| --- | --- | --- | --- |
| **Distance between Decision Thresholds (α)** | 2.2 95% CI [2.11; 2.298] | 2.451 95% CI [2.326; 2.58] | 2.458 95% CI [2.35; 2.573] |
| **Non-Decision Time (t0)** | 6.973 95% [6.799; 7.139] | 6.685 95% CI [6.429; 6.949] | 6.757 95% CI [6.541; 6.973] |
| **Starting Point (z)** | 0.496 95% CI [0.484; 0.504] | 0.477 95% CI [0.467; 0.487] | 0.472 95% CI [0.463; 0.482] |
| **Drift Rate (v)** | 0.11 95% CI [0.048; 0.171] | 0.198 95% CI [0.147; 0.248] | 0.287  95% CI [0.242; 0.335] |
