# Supplementary material for: Changing the incentive structure of social media platforms to halt the spread of misinformation
Source: eLife. 2023 Jun 6;12:e85767. doi: 10.7554/eLife.85767 (PMC10259455; doi:10.7554/eLife.85767)
Supplement: Supplementary file 16. [file elife-85767-supp16.docx]

**Supplementary file 16. Mean difference in posterior distributions and 95% HDI Comparison in Experiment 5.**

| **Estimate** | **‘(Dis)Trust’ minus Baseline** | **‘(Dis)Trust’ minus ‘(Dis)Like’** | **‘(Dis)Like’ minus Baseline** |
| --- | --- | --- | --- |
| **Distance between Decision Thresholds (α)** | 0.258 [0.101 0.415] | 0.007 [-0.17; 0.186] | 0.251 [0.085; 0.418] |
| **Non-Decision Time (t0)** | -0.216 [-0.502; 0.07] | 0.072 [-0.293; 0.432] | -0.288 [-0. 622; 0.046] |
| **Starting Point (z)** | -0.024 [-0.04; -0.007] | -0.005 [-0.019; 0.009] | -0.019 [-0.036; -0.002] |
| **Drift Rate (v)** | 0.177 [0.095; 0.259] | 0.089 [0.018; 0.163] | 0.088 [0.005; 0.173] |
