# Supplementary material for: Changing the incentive structure of social media platforms to halt the spread of misinformation
Source: eLife. 2023 Jun 6;12:e85767. doi: 10.7554/eLife.85767 (PMC10259455; doi:10.7554/eLife.85767)
Supplement: Supplementary file 17. [file elife-85767-supp17.docx]

**Supplementary file 17. Group estimates for DDM in Experiment 6.**

| **Estimate** | **Baseline** | **‘(Dis)Like’** | **‘(Dis)Trust’** |
| --- | --- | --- | --- |
| **Distance between Decision Thresholds (α)** | 2.21 95% CI [2.117; 2.309] | 2.489 95% CI [2.298; 2.691] | 2.461 95% CI [2.312; 2.619] |
| **Non-Decision Time (t0)** | 6.982 95% [6.812; 7.151] | 6.476 95% CI [6.172; 6.769] | 6.819 95% CI [6.567; 7.063] |
| **Starting Point (z)** | 0.493 95% CI [0.482; 0.505] | 0.483 95% CI [0.47; 0.497] | 0.469 95% CI [0.458; 0.48] |
| **Drift Rate (v)** | 0.139 95% CI [0.065; 0.215] | 0.172 95% CI [0.112; 0.233] | 0.321 95% CI [0.265; 0.379] |
