# Supplementary material for: Changing the incentive structure of social media platforms to halt the spread of misinformation
Source: eLife. 2023 Jun 6;12:e85767. doi: 10.7554/eLife.85767 (PMC10259455; doi:10.7554/eLife.85767)
Supplement: Supplementary file 18. [file elife-85767-supp18.docx]

**Supplementary file 18. Mean difference in posterior distributions and 95% HDI Comparison in Experiment 6.**

| **Estimate** | **‘(Dis)Trust’ minus Baseline** | **‘(Dis)Trust’ minus ‘(Dis)Like’** | **‘(Dis)Like’ minus Baseline** |
| --- | --- | --- | --- |
| **Distance between Decision Thresholds (α)** | 0.251 [0.059; 0.442] | -0.027 [-0.29; 0.227] | 0.278 [0.048; 0.511] |
| **Non-Decision Time (t0)** | -0.163 [-0.491; 0.157] | 0.343 [-0.059; 0.749] | -0.506 [-0. 859; -0.152] |
| **Starting Point (z)** | -0.025 [-0.041; -0.008] | -0.014 [-0.033; 0.005] | -0.011 [-0.029; 0.008] |
| **Drift Rate (v)** | 0.182 [0.085; 0.28] | 0.149 [0.058; 0.236] | 0.033 [-0.068; 0.135] |
