# Supplementary material for: Changing the incentive structure of social media platforms to halt the spread of misinformation
Source: eLife. 2023 Jun 6;12:e85767. doi: 10.7554/eLife.85767 (PMC10259455; doi:10.7554/eLife.85767)
Supplement: Supplementary file 19. [file elife-85767-supp19.docx]

**Supplementary file 19. Recovered Group estimates for DDM in Experiment 2 based on simulated data.**

| **Estimate** | **Baseline** | **‘(Dis)Like’** | **‘(Dis)Trust’** |
| --- | --- | --- | --- |
| **Distance between Decision Thresholds (α)** | 2.143 95% CI [2.11; 2.176] | 2.336  95% CI [2.314; 2.358] | 2.379  95% CI [2.35; 2.41] |
| **Non-Decision Time (t0)** | 7.016 95% CI [6.986; 7.046] | 6.945 95% CI [6.929; 6.962] | 6.667  95% CI [6.648; 6.687] |
| **Starting Point (z)** | 0.499  95% CI [0.479; 0.518] | 0.501  95% CI [0.485; 0.517] | 0.486  95% CI [0.468; 0.504] |
| **Drift Rate (v)** | 0.107  95% CI [0.068; 0.146] | 0.107 95% CI [0.081; 0.133] | 0.24  95% CI [0.209; 0.27] |
