# Supplementary material for: Changing the incentive structure of social media platforms to halt the spread of misinformation
Source: eLife. 2023 Jun 6;12:e85767. doi: 10.7554/eLife.85767 (PMC10259455; doi:10.7554/eLife.85767)
Supplement: Supplementary file 20. [file elife-85767-supp20.docx]

**Supplementary file 20. Recovered Group estimates for DDM in Experiment 3 based on simulated data.**

| **Estimate** | **Baseline** | **‘(Dis)Like’** | **‘(Dis)Trust’** |
| --- | --- | --- | --- |
| **Distance between Decision Thresholds (α)** | **2.247 95% CI [2.209; 2.284]** | **2.213 95% CI [2.183; 2.243]** | **2.195**  **95% CI [2.163; 2.228]** |
| **Non-Decision Time (t0)** | **6.904 95% [6.884; 6.926]** | **7.04 95% CI [7.021; 7.059]** | **7.083**  **95% CI [7.061; 7.105]** |
| **Starting Point (z)** | **0.493 95% CI [0.475; 0.511]** | **0.515 95% CI [0.498; 0.531]** | **0.485**  **95% CI [0.468; 0.501]** |
| **Drift Rate (v)** | **-0.005 95% CI [-0.035; 0.024]** | **0.007 95% CI [-0.024; 0.036]** | **0.141**  **95% CI [0.1; 0.181]** |
