# Supplementary material for: Changing the incentive structure of social media platforms to halt the spread of misinformation
Source: eLife. 2023 Jun 6;12:e85767. doi: 10.7554/eLife.85767 (PMC10259455; doi:10.7554/eLife.85767)
Supplement: Supplementary file 21. [file elife-85767-supp21.docx]

**Supplementary file 21. Pairwise Comparisons for Discernment Experiment 2.**

| **Pairwise Comparison** | **Experimental Data** | | | **Simulated Data** | | |
| --- | --- | --- | --- | --- | --- | --- |
|  | **Mean 1 (SE)** | **Mean 2 (SE)** | **Statistic** | **Mean 1 (SE)** | **Mean 2 (SE)** | **Statistic** |
| **(Dis)Trust**  **vs**  **Baseline** | 0.18 (0.018) | 0.109 (0.028) | t(152)=3.112, p=0.002,  Cohen’s d=0.515 | 0.2 (0.027) | 0.109 (0.028) | t(152)=2.243, p=0.026,  Cohen’s d=0.372 |
| **(Dis)Trust**  **vs**  **(Dis)Like** | 0.18 (0.018) | 0.085 (0.019) | t(227)=3.464, p<0.001,  Cohen’s d=0.465 | 0.2 (0.027) | 0.118 (0.022) | t(227)=2.407, p=0.017,  Cohen’s d=0.323 |
| **(Dis)Like**  **vs**  **Baseline** | 0.085 (0.019) | 0.084 (0.025) | t(191)=0.007, p=0.995,  Cohen’s d=0.001 | 0.118 (0.022) | 0.109 (0.028) | t(191)=0.255, p=0.822,  Cohen’s d=0.035 |
