# Supplementary material for: Changing the incentive structure of social media platforms to halt the spread of misinformation
Source: eLife. 2023 Jun 6;12:e85767. doi: 10.7554/eLife.85767 (PMC10259455; doi:10.7554/eLife.85767)
Supplement: Supplementary file 22. [file elife-85767-supp22.docx]

**Supplementary file 22. Pairwise Comparisons for Discernment Experiment 3.**

| **Pairwise Comparison** | **Experimental Data** | | | **Simulated Data** | | |
| --- | --- | --- | --- | --- | --- | --- |
|  | **Mean 1 (SE)** | **Mean 2 (SE)** | **Statistic** | **Mean 1 (SE)** | **Mean 2 (SE)** | **Statistic** |
| **(Dis)Trust**  **vs**  **Baseline** | 0.101 (0.015) | 0.008 (0.014) | t(261)=4.498, p<0.001, Cohen’s d=0.555 | 0.116 (0.024) | -0.019 (0.025) | t(261)=3.826, p<0.001, Cohen’s d=0.285 |
| **(Dis)Trust**  **vs**  **(Dis)Like** | 0.101 (0.015) | 0.042 (0.013) | t(263)=2.958, p=0.003, Cohen’s d=0.364 | 0.116 (0.024) | 0.032 (0.024) | t(263)=2.463, p=0.014, Cohen’s d=0.303) |
| **(Dis)Like**  **vs**  **Baseline** | 0.042 (0.013) | 0.008 (0.014) | t(252)=1.731, p=0.085, Cohen’s d=0.217 | 0.032 (0.024) | -0.019 (0.025) | t(252)=1.476, p=0.141, Cohen’s d=0.185 |
