# Supplementary material for: Changing the incentive structure of social media platforms to halt the spread of misinformation
Source: eLife. 2023 Jun 6;12:e85767. doi: 10.7554/eLife.85767 (PMC10259455; doi:10.7554/eLife.85767)
Supplement: Supplementary file 23. [file elife-85767-supp23.docx]

**Supplementary file 23. Correlations between participants’ real and recovered DDM estimates in Experiment 2 and Experiment 3.**

| **Estimate** | **Experiment 2** | **Experiment 3** |
| --- | --- | --- |
| **Distance between Decision Thresholds (α)** | *r* = 0.926, *p* < 0.001 | *r* = 0.886, *p*<0.001 |
| **Non-Decision Time (t0)** | *r* = 0.997, *p*<0.001 | *r* = 0.995, *p<0.001* |
| **Starting Point (z)** | *r* = 0.471, *p*<0.001 | *r* = 0.321, *p<0.001* |
| **Drift Rate (v)** | *r* = 0.869, *p*<0.001 | *r* = 0.877, *p*<0.001 |

We estimated both group-level and individual-level parameters. We then used the individual-level parameter estimates to simulate data for each participant respectively in the dataset. We used the same number of trials as in the experiments. Simulated data from each participant were then combined and used to perform model recovery analysis. We sampled 2000 times from the posteriors, discarding the first 500 as burn in. We then correlated the real and the recovered individual-level parameters.
