# Supplementary material for: Changing the incentive structure of social media platforms to halt the spread of misinformation
Source: eLife. 2023 Jun 6;12:e85767. doi: 10.7554/eLife.85767 (PMC10259455; doi:10.7554/eLife.85767)
Supplement: Supplementary file 24. [file elife-85767-supp24.docx]

**Supplementary file 24. Individual Ratings per Stimulus in Experiment 1.**

| **Stimuli** | **Veracity** | **Trusts** | **Distrusts** | **Likes** | **Dislikes** | **Skips** |
| --- | --- | --- | --- | --- | --- | --- |
| 90% to 95% of those hospitalized for COVID-19 in the United States are unvaccinated. | TRUE | 53 | 29 | 20 | 17 | 12 |
| Over,the past two years, climate and weather disaster damage has cost the US over 400 billion dollars. | TRUE | 63 | 12 | 22 | 28 | 18 |
| The concentration of carbon dioxide in the earth’s atmosphere has climbed to a level last seen more than 3 million years ago. | TRUE | 38 | 26 | 12 | 35 | 25 |
| Flamingos dye their sun-faded feathers to attract mates. | TRUE | 22 | 37 | 47 | 4 | 26 |
| Climate change has made hurricanes more dangerous, but not more frequent. | TRUE | 34 | 34 | 19 | 18 | 26 |
| Babies born in 2020 may suffer up to 7 times as many extreme heatwaves as 1960s kids. | TRUE | 47 | 25 | 9 | 42 | 22 |
| Some dinosaurs may have lived in herds as early as 193 million years ago. | TRUE | 48 | 11 | 53 | 4 | 21 |
| Some birds learn to recognize calls while still in their eggs. | TRUE | 44 | 8 | 75 | 1 | 13 |
| Wild parsnips can cause skin blisters which are dangerous to humans. | TRUE | 40 | 19 | 20 | 19 | 36 |
| A canadian woman was nearly hit by a meteorite that crashed through her bedroom ceiling. | TRUE | 41 | 19 | 36 | 12 | 24 |
| A solar storm hit the earth and brought northern lights to New York. | TRUE | 31 | 33 | 51 | 3 | 18 |
| Climate change is making the earth dimmer. | TRUE | 21 | 43 | 10 | 23 | 35 |
| Rain fell at the normally snowy summit of Greenland for the first time on record. | TRUE | 40 | 20 | 25 | 19 | 26 |
| A third of Antarctic ice shelf risks collapse as our planet warms. | TRUE | 63 | 15 | 14 | 42 | 15 |
| The clothing industry contributes up to 10% to the pollution driving the climate crisis. | TRUE | 57 | 11 | 13 | 36 | 20 |
| Deforestation has made humans more vulnerable to pandemics. | TRUE | 36 | 32 | 16 | 28 | 30 |
| From 2010 to 2017, natural gas production decreased by nearly 70% in New York and increased almost 1000%. | TRUE | 23 | 33 | 8 | 13 | 49 |
| Each year, 324,000 pregnant women experience domestic violence during their pregnancy. | TRUE | 57 | 7 | 5 | 64 | 15 |
| San Francisco had twice as many drug overdose deaths as COVID deaths last year. | TRUE | 38 | 26 | 8 | 39 | 21 |
| Marijuana intake is significantly correlated to psychotic disorders, particularly in teenagers. | TRUE | 30 | 43 | 11 | 34 | 19 |
| Overdose deaths in West Virginia are up by 45% from the prior year. | TRUE | 45 | 11 | 8 | 54 | 19 |
| Tattoo ink isn’t approved by the U.S. Food and Drug Administration. | TRUE | 22 | 32 | 7 | 26 | 39 |
| The fortification of flour with folic acid can prevent certain birth defects. | TRUE | 27 | 33 | 34 | 7 | 32 |
| Replacing table salt with a low-sodium substitute lowers the risk of stroke and other cardiovascular diseases. | TRUE | 56 | 12 | 57 | 4 | 14 |
| Professional soccer defenders, who head the ball most often, are almost five times more likely to develop a brain disease than the average person. | TRUE | 48 | 22 | 12 | 30 | 22 |
| Some fast-food items contain plastics linked to serious health problems. | TRUE | 45 | 19 | 18 | 32 | 23 |
| In the last 10 years less than half of the adults in the U.S. received a flu shot. | TRUE | 48 | 20 | 18 | 22 | 21 |
| Burnt seeds show that people used tobacco 12,000 years ago. | TRUE | 45 | 11 | 41 | 5 | 27 |
| Twitter is banned in Iran. | TRUE | 43 | 11 | 15 | 30 | 33 |
| France sets a minimum book delivery fee in effort to protect independent stores from Amazon. | TRUE | 37 | 13 | 56 | 4 | 28 |
| 82% of gun owners in the U.S. support requiring all gun buyers to pass a background check. | TRUE | 49 | 16 | 68 | 4 | 12 |
| The US is the only modern industrialized country that does not already have a paid family medical leave. | TRUE | 55 | 15 | 11 | 48 | 19 |
| Canada charges the U.S. a 270% tariff on dairy products. | TRUE | 17 | 36 | 10 | 37 | 25 |
| China imposes the death penalty on drug dealers. | TRUE | 39 | 10 | 20 | 29 | 31 |
| The Texas power grid is not part of the U.S. power grid because Texas wanted to avoid federal regulation. | TRUE | 46 | 30 | 15 | 23 | 26 |
| Black-Lives-Matter apparels and other political expressions were banned at the 2021 Olympics. | TRUE | 23 | 30 | 22 | 32 | 26 |
| Having higher testosterone levels generally provides an advantage in athletic performance. | TRUE | 64 | 13 | 39 | 9 | 17 |
| Astronomers may have spotted the first known exoplanet in another galaxy. | TRUE | 44 | 9 | 66 | 1 | 21 |
| Surgeons in New York City successfully attached a pig kidney to a human patient. | TRUE | 50 | 17 | 39 | 5 | 29 |
| China’s lunar rock samples show that lava flowed on the moon 2 billion years ago. | TRUE | 27 | 27 | 40 | 4 | 31 |
| All identical twins may share a common set of chemical markers on their DNA. | TRUE | 62 | 4 | 48 | 0 | 23 |
| Scientists used a tiny brain implant to help a blind teacher see letters again. | TRUE | 42 | 17 | 69 | 2 | 13 |
| 30 years ago, the US. ranked sixth among advanced economies in the share of women in the workforce. Today they rank 23th. | TRUE | 26 | 37 | 12 | 29 | 29 |
| Approximately 60,000 Canadians currently live undocumented in the USA. | TRUE | 29 | 29 | 17 | 18 | 38 |
| Rwanda presently has the global highest number of women in parliament at 61.1%. | TRUE | 36 | 9 | 63 | 3 | 26 |
| More than a million Californians do not have direct access to clean water. | TRUE | 33 | 33 | 5 | 40 | 21 |
| Inflation is costing US households with medium incomes an extra $175 a month. | TRUE | 70 | 8 | 16 | 54 | 9 |
| People of color and those of not use marijuana at the same rate. People of color are 3 times more likely to get arrested and convicted. | TRUE | 58 | 13 | 14 | 36 | 24 |
| 10% of children in the United States are Texans. | TRUE | 21 | 30 | 16 | 7 | 43 |
| More than a half of Afghanistan’s population faces an acute food crisis this winter. | TRUE | 62 | 2 | 10 | 48 | 15 |
| Reports show that babies whose mothers got the COVID-19 vaccine died after breastfeeding. | FALSE | 3 | 92 | 2 | 47 | 11 |
| In the United Kingdom, 81% of COVID- 19 deaths are among the vaccinated. | FALSE | 15 | 75 | 5 | 34 | 14 |
| United Airlines lost 40% of their staff because of vaccine requirements. | FALSE | 46 | 31 | 13 | 42 | 13 |
| COVID-19 vaccines contain graphene and living organisms made of aluminum. | FALSE | 6 | 70 | 3 | 30 | 29 |
| At least two children died in Australia after receiving COVID-19 vaccines in a mass vaccination program for students. | FALSE | 31 | 37 | 2 | 45 | 18 |
| Test results show that Covid-19 shots damage the immune system. | FALSE | 14 | 80 | 4 | 36 | 12 |
| Children are more likely to end up in the hospital because of the vaccine than they are because of COVID | FALSE | 15 | 75 | 7 | 49 | 12 |
| Vaccinated individuals are more susceptible to COVID variant infections than unvaccinated. | FALSE | 13 | 80 | 5 | 38 | 12 |
| Cancer increased twentyfold among COVID-19 vaccinated due to suppressed T cells. | FALSE | 12 | 78 | 0 | 36 | 16 |
| COVID-19 PCR tests cannot differentiate between flu and COVID-19. | FALSE | 10 | 73 | 7 | 31 | 14 |
| A wind turbine could never generate as much energy as was invested in building it. | FALSE | 27 | 59 | 13 | 31 | 12 |
| 90 percent of the world’s glaciers are growing. | FALSE | 11 | 69 | 18 | 17 | 25 |
| Forest fires are caused by poor management. Not by climate change. | FALSE | 26 | 55 | 17 | 29 | 18 |
| Ivermectin sterilizes the majority (85%) of the men who take it. | FALSE | 7 | 79 | 6 | 24 | 20 |
| The Centers for Disease Control and Prevention warn of a polio-like outbreak in children coming within the next four months. | FALSE | 15 | 59 | 4 | 30 | 19 |
| It would cost $20 billion to end homelessness in the U.S. and halting global warming would cost $300 billion. | FALSE | 34 | 33 | 24 | 14 | 25 |
| An air quality test under a mask proved that it is not healthy to wear one. | FALSE | 19 | 69 | 15 | 35 | 13 |
| Flu cases dropped by 379 million in one year. | FALSE | 31 | 37 | 37 | 8 | 24 |
| New York hospitals reported thousands of fungal lung infections from mask- wearing. | FALSE | 15 | 70 | 5 | 40 | 15 |
| The Impossible Burger contains more estrogen than transgender hormone therapy. | FALSE | 13 | 73 | 4 | 27 | 20 |
| Prenatal ultrasounds carry extreme risks, including miscarriage and genetic damage. | FALSE | 5 | 89 | 2 | 32 | 14 |
| For men, a positive pregnancy test equals testicular cancer. | FALSE | 13 | 73 | 8 | 26 | 20 |
| AIDS was cured in more than a dozen patients. | FALSE | 26 | 37 | 46 | 7 | 22 |
| Abortion increases the risk of breast cancer. | FALSE | 12 | 72 | 6 | 41 | 17 |
| It is possible to completely detox the body from chemicals. | FALSE | 18 | 62 | 32 | 14 | 13 |
| Two forms of fluoride in our drinking water are labeled as extremely toxic by the Centers for Disease Control and Prevention. | FALSE | 29 | 48 | 9 | 37 | 20 |
| Sophia Stewart wrote books in the 70s that were stolen from her by Warner Bros. She won Hollywood‘s biggest lawsuit. | FALSE | 22 | 14 | 37 | 11 | 48 |
| Gravestones in Japanese cemeteries have QR-codes which can be scanned to get a biography of the deceased person. | FALSE | 21 | 33 | 44 | 13 | 24 |
| 52% of Metropolitan Police officers have been found guilty of sexual misconduct while wearing uniform in the line of duty. | FALSE | 20 | 55 | 1 | 47 | 16 |
| Most of the money made by the National Football League goes to the players. | FALSE | 17 | 61 | 14 | 18 | 22 |
| The Biden administration gifted the Taliban with over $80 billion worth of military grade weapons. | FALSE | 31 | 53 | 3 | 52 | 11 |
| Sweden is abolishing cash. | FALSE | 18 | 29 | 21 | 27 | 35 |
| Refugees get more in monthly benefits than social security recipients. | FALSE | 24 | 47 | 9 | 48 | 12 |
| Members of Congress and their families and staff are exempt from repaying student loans. | FALSE | 15 | 38 | 7 | 58 | 23 |
| A single immigrant can bring an unlimited number of relatives to the U.S. | FALSE | 15 | 61 | 6 | 37 | 18 |
| The Soviet Union took all of its equipment from Afghanistan. | FALSE | 9 | 62 | 6 | 19 | 32 |
| There wasn’t a single American casualty in Afghanistan in the last year and a half of the Trump administration. | FALSE | 23 | 60 | 26 | 13 | 19 |
| More than half of the human genes are identical to those of mice. | FALSE | 43 | 30 | 23 | 7 | 29 |
| The top 1% pays 90% of income taxes in the U.S. | FALSE | 18 | 69 | 10 | 30 | 12 |
| The American murder rate is 50 times that of any other developed nation. | FALSE | 42 | 28 | 8 | 49 | 14 |
| The U.S. poverty rate is the 4th highest in the world. | FALSE | 37 | 33 | 5 | 45 | 20 |
| In the United States, 50 percent of social services are provided by the Catholic church. | FALSE | 13 | 52 | 10 | 17 | 35 |
| Marine fossils found on the Mount Everest are evidence of a global flooding. | FALSE | 35 | 23 | 41 | 6 | 32 |
| Joe Biden’s climate plan includes cutting 90% of red meat from our diets by 2030. | FALSE | 9 | 65 | 12 | 51 | 10 |
| 14,000 abandoned wind turbines litter the United States. | FALSE | 29 | 29 | 9 | 49 | 22 |
| Wildfires were worse in the early part of the 1900s than they are today. | FALSE | 16 | 59 | 3 | 18 | 30 |
| The U.S. corn crop, at its peak, produces 40% more oxygen than the Amazon rainforest. | FALSE | 21 | 41 | 39 | 6 | 25 |
| An electric car costs more than seven times as much as a gasoline powered car. | FALSE | 22 | 57 | 7 | 32 | 21 |
| The amount of coral on the Great Barrier Reef is at record high levels. | FALSE | 56 | 35 | 10 | 19 | 22 |
| There has not been a long-term distinctive change in sea level rise rates in the last 120 years. | FALSE | 62 | 14 | 24 | 21 | 25 |
